# The Response to Oxidative Stress in *Listeria monocytogenes* Is Temperature Dependent

**DOI:** 10.3390/microorganisms8040521

**Published:** 2020-04-05

**Authors:** Beatriz Manso, Beatriz Melero, Beatrix Stessl, Isabel Jaime, Martin Wagner, Jordi Rovira, David Rodríguez-Lázaro

**Affiliations:** 1 Department of Biotechnology and Food Science, Faculty of Sciences, University of Burgos, 09001 Burgos, Spain; beatrizmg@ubu.es (B.M.); ijaime@ubu.es (I.J.); jorvira@ubu.es (J.R.); 2 Institute of Milk Hygiene, Milk Technology and Food Science, Department for Farm Animals and Veterinary Public Health, University of Veterinary Medicine, A-1210 Vienna, Austria; Beatrix.Stessl@vetmeduni.ac.at (B.S.); Martin.Wagner@vetmeduni.ac.at (M.W.); 3 Christian Doppler Laboratory for Molecular Food Analytics, University of Veterinary Medicine, A-1210 Vienna, Austria

**Keywords:** pathogen, virulence, survival, food industry, oxidizing agents, gene expression

## Abstract

The stress response of 11 strains of *Listeria monocytogenes* to oxidative stress was studied. The strains included ST1, ST5, ST7, ST6, ST9, ST87, ST199 and ST321 and were isolated from diverse food processing environments (a meat factory, a dairy plant and a seafood company) and sample types (floor, wall, drain, boxes, food products and water machine). Isolates were exposed to two oxidizing agents: 13.8 mM cumene hydroperoxide (CHP) and 100 mM hydrogen peroxide (H_2_O_2_) at 10 °C and 37 °C. Temperature affected the oxidative stress response as cells treated at 10 °C survived better than those treated at 37 °C. H_2_O_2_ at 37 °C was the condition tested resulting in poorest *L. monocytogenes* survival. Strains belonging to STs of Lineage I (ST5, ST6, ST87, ST1) were more resistant to oxidative stress than those of Lineage II (ST7, ST9, ST199 and ST321), with the exception of ST7 that showed tolerance to H_2_O_2_ at 10 °C. Isolates of each ST5 and ST9 from different food industry origins showed differences in oxidative stress response. The gene expression of two relevant virulence (*hly*) and stress (*clp*C) genes was studied in representative isolates in the stressful conditions. *hly* and *clp*C were upregulated during oxidative stress at low temperature. Our results indicate that conditions prevalent in food industries may allow *L. monocytogenes* to develop survival strategies: these include activating molecular mechanisms based on cross protection that can promote virulence, possibly increasing the risk of virulent strains persisting in food processing plants.

## 1. Introduction

The bacterium *Listeria monocytogenes* is ubiquitous, and able to survive and grow at a wide range of temperatures, and in alkaline or acid media and high osmolality conditions [1]. Most of these stressful conditions are common in food processing environments (FPE) and inside the human host during infection [2]. *L. monocytogenes* is exposed to acid and high osmolality within food matrices (e.g. in dairy products after fermentation or in brine tanks and after addition of food preservatives) [3]. Likewise, gastric acid provides a harsh environment [4].

*L. monocytogenes* is exposed to diverse stresses in FPEs and during infection. Refrigeration to preserve food products both in production facilities and in consumers’ fridges imposes low temperatures, and oxidative stress is caused by sanitizer agents, especially disinfectant application and antibiotic treatments [5,6]. Disinfectants based on quaternary ammonium compounds are the most common bactericidal agents used in the food industry, and chlorine derivates or peracetic acid are also applied to prevent *L. monocytogenes* spread within facilities [7]. Hydrogen peroxide (H_2_O_2_) is a non-toxic, hydro-soluble and bacteriostatic or bactericidal agent also commonly used as a disinfectant [8]. Oxidizing agents cause several types of damage in cells, affecting the peptidoglycan wall and cell membrane, denaturing proteins and disrupting nucleic acid structure [6,9]. *L. monocytogenes* can sense stressful conditions through molecular signalling [10] and activates survival strategies to reduce oxidative damage; these strategies include expression of *sig*B, cold and heat shock proteins (*csp*ABCD), proteases (*clp*C, *clp*P, *gro*EL) and genes related to oxidative response notably superoxide dismutase (*sod*), *per*R and catalase (*kat*) [11,12,13]. *sig*B acts on genes related to stress (GRS) and virulence genes such as *inl*A and LIPI-1 [5]. *L. monocytogenes* virulence can increase under stress conditions: *prf*A is regulated by a *sig*B-depedent promoter, and *clp*C expression influences some genes responsible for adherence [13,14]. This relation between virulence and the stress response illustrates how *L. monocytogenes* may protect itself in different stressful conditions, being able to survive in environments with multiple stress factors [15].

The first aim of this study was to analyse the effect of oxidizing agents on the growth of *L. monocytogenes* at optimal and refrigeration temperatures. The second was to study changes in *hly* and *clp*C expression to investigate the relationship between virulence and the oxidative stress response.

## 2. Materials and Methods

### 2.1. Bacterial and Culture Conditions

Table 1 collects the information of the eleven strains used in this study that were previously isolated and characterized in Manso et al. and Melero et al. [3,16,17]. The strains of *L. monocytogenes* belonged to eight sequence types (ST) (ST1 (*n* = 1), ST5 (*n* = 2), ST6 (*n* = 1), ST7 (*n* = 1), ST9 (*n* = 3), ST87 (*n* = 1), ST199 (*n* = 1) and ST321 (*n* = 1)). Moreover, they were isolated from three food processing plants: six strains from a poultry meat factory, four from a dairy plant and one from a seafood company. They were found on non-food contact surfaces (*n* = 6), food contact surfaces (*n* = 2) and food (*n* = 3) samples. They were grown on Chromogenic Listeria Agar ISO (Oxoid, United Kingdom) at 37 °C for 48 hours. One single colony from each OCLA plates was streaked onto Tryptone Soya Agar (TSA, Oxoid) plates supplemented with 0.6% yeast extract (YE, Pronadisa, Madrid, Spain) and incubated at 37 °C for 24 hours. A single colony from each plate was used to inoculate 5 mL of Brain Heart Infusion broth (BHI broth) (Oxoid) and incubated statically overnight at 37 °C. 

### 2.2. Oxidative Stress Assay

*L. monocytogenes* strains were grown in RPMI broth medium (1× RPMI-1640 Medium, HyClone^™^ and 2.05 mM L-Glutamine, GE Healthcare Life Sciences) [18] supplemented with oxidative agents according to Rea et al. [19] but with some modifications. Approximately 10^9^ cfu/mL of the overnight culture was inoculated to 50 mL fresh medium (BHI broth) and incubated until mid-exponential phase (OD_600_ ~ 0.8) with shaking. After that, the overnight culture was distributed in 10 mL and centrifuged at 14,600× *g* for 5 min at room temperature. The bacterial pellets were collected, washed with Ringer solution (Oxoid), and centrifuged again as previously. The pellets were then resuspended in 10 mL of RPMI medium containing 8 mg/mL ferric citrate (Sigma, San Luis, Misuri, USA) and 13.8 mM cumene hydroperoxide (CHP) (Aldrich) [20], or 100 mM hydrogen peroxide (H_2_O_2_) (VWR Chemicals), or with no added agent (controls). These *Listeria* cultures were incubated at 10 °C and 37 °C for 4 hours, and two aliquots were taken after 2, 3 and 4 hours for enumeration and RNA extraction. Serial decimal dilutions were streaked onto TSAYE plates and were incubated at 37 °C for 24 hours to calculate the susceptibility of *L. monocytogenes* strains against oxidative stress conditions. All the experiments were performed in triplicate.

### 2.3. RNA Extraction and Gene Expression

Five out of the eleven *L. monocytogenes* strains (ST87, ST5 and ST9 -from a meat industry-; ST9 from a dairy plant and the ST321 from a seafood company) were chosen to perform RNA extraction using the RNA Pure Link^™^ RNA Mini Kit (Invitrogen, Carlsbad, California, USA) following the manufacturer’s recommendations (Table 1). RNA samples were reverse transcribed using the ImProm-II^™^ Reverse Transcription System (Promega, USA) as described previously [21]. Resulting cDNAs were diluted 1:20 and used as templates for specific real-time PCR assays as previously described [21] in a StepOne Real-Time PCR System (Applied Biosystems, Foster City, California, USA). Expression of *hly* (listeriolysin O gene) [22] and *clp*C (endopeptidase Clp ATP binding chain gene) [23] was studied and *ldh* (lactate dehydrogenase gene) [21] was used for normalization results following the 2^-∆∆Ct^ quantification method.

### 2.4. Statistical Analysis

A multifactor analysis of variance was used to determine the correlation between the response to each temperature and oxidizing agents in all *L. monocytogenes* strains. Fisher’s least significant difference (LSD) procedure was used to determine any significant differences (*p* values < 0.05) amongst the means between the results for the oxidative stress at 37 °C and that at 10 °C. (Stat Graphics Centurion XVI software, Stat Graphics Centurion, Madrid, Spain).

## 3. Results

### 3.1. Response to Oxidative Stress

Table 1 shows the results of the oxidative stress in the *L. monocytogenes* strains tested. *L. monocytogenes* strains, regardless of their origin or genetic background, were significantly (*p* < 0.05) more tolerant to oxidizing agents (CHP and H_2_O_2_) at 10 ºC than at 37 ºC. The stress response was also significantly different (*p* < 0.05) between CHP and H_2_O_2_, and the mean H_2_O_2_ effect was significantly higher (*p* < 0.05) (Table 1). 

The response to oxidative stress differed between strains at the same temperature depending on the food industry origin (Table 1). The oxidative stress response to CHP at 37 ºC differed between ST5 and ST9 strains depending on the sample types and site of isolation (Table 1) although the differences overall between strains at 37 °C were not significant (*p* = 1). The ST9 strain isolated from a floor in a meat processing plant was more resistant to CHP at 37 °C during the first hour (reduction of 6.07 log units); however, ST9 strains isolated from a wall in the same meat factory and from cheese crumbs showed higher count reductions (6.75 and 7.23 log unit, respectively) (Table 1). Similarly, the count reduction during the first hour for the ST5 strain isolated from the meat processing plant was lower than that for the ST5 strain isolated from the dairy plant (5.17 vs 5.70 log units) and it continued to survive after 3 h (Table 1). The ST321 strain from the seafood facility and the ST9 strain from the meat factory wall were not detectable after 2 h of incubation, whereas strains from the meat processing plant, belonging to ST1 (7.88 log unit decline), ST87 (8.20 log unit decline) and ST5 (9 log unit decline), survived for 3 h (Table 1). Similarly, the stress response to CHP at 10 °C was different within ST5 and ST9 strains; the reduction for the isolates from cheese crumbs was lower than those for the isolates from the meat processing plant: 1.93 and 2.24 log unit reduction vs. 2.35 and 2.60 and 3.18 log unit reduction after 3h of incubation, respectively (Table 1). The LSD Test indicated that the ST9 strain from the wall sample (meat processing) and the ST199 strain were significantly the most susceptible (*p* = 0.0129) to all the other strains at 10 °C; both were the most susceptible strains at refrigeration temperature. By contrast, ST1 and ST6 strains were the most resistant to CHP at 10 °C (Table 1). 

Similar to our observations for CHP, lower temperature moderated the effect of the oxidative stress; *L. monocytogenes* strains were more tolerant to H_2_O_2_ at 10 °C than at 37 °C regardless the origin or genetic background of the strains. The oxidative stress response in *L. monocytogenes* to H_2_O_2_ at 37 °C was higher than to CHP. No colonies were found after just 1 hour of incubation in H_2_O_2_ at 37 °C, except for the ST5 strain from the dairy plant and ST87 −7.26 and 6.67 log unit declines, respectively- (Data not shown). However, after incubation at 10 °C for 3 hours, H_2_O_2_ was less toxic than CHP for the *L. monocytogenes* strains: count reductions were between 1.20 (ST87) and > 9.30 (ST9) log units (Table 1).

### 3.2. Gene Expression in Oxidative Stress Conditions

**Figure 1:**
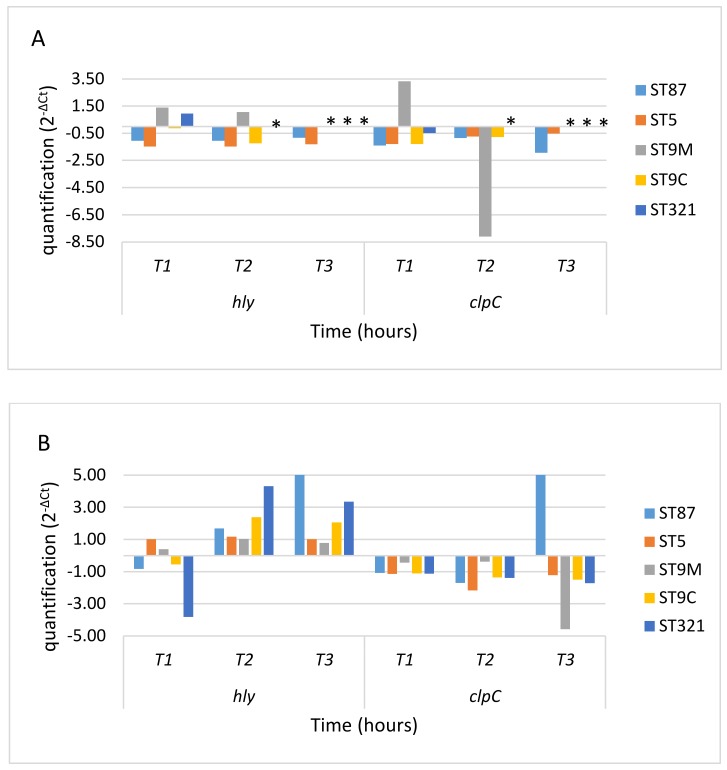

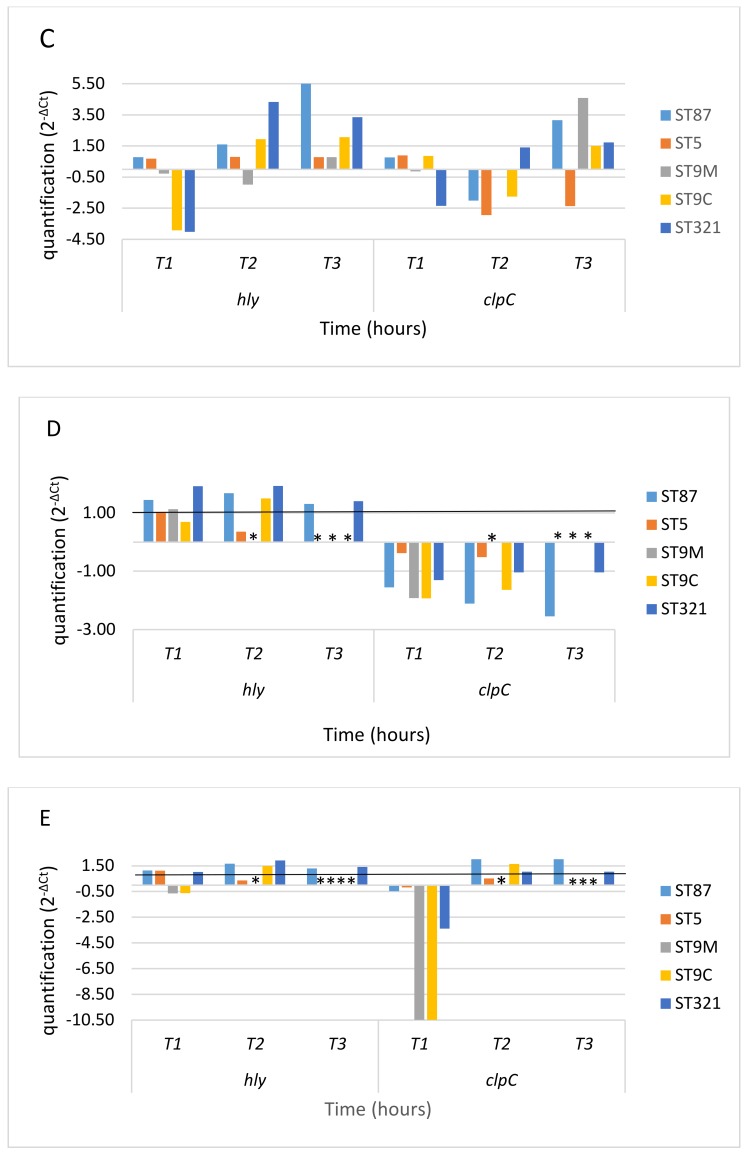
Expression of *hly* and *clp*C genes during oxidative stress. Transcripts of *hly* and *clp*C were normalized to those of the *ldh* gene. Expression of *hly* and *clp*C after 1, 2 and 3 hours is shown relative to that before the addition of the oxidizing agents. (**A**) Exposure to CHP at 37 °C; (**B**) Exposure to CHP at 10 °C; (**C**) Expression of *hly* and *clp*C in CHP at 10 °C relative to that at 37 °C; (**D**) Exposure to H_2_O_2_ at 10 °C; and (**E**) Expression of *hly* and *clp*C in H_2_O_2_ at 10 °C relative to that at 37 °C. Black line: No differences in gene expression with respect T_0_ (value = 1). *: gene expression results were not studied because cell counts were below the detection limit. T1, T2 and T3: period of time after 1 hour (T1), 2 hours (T2) and 3 hours (T3) of oxidative stress conditions exposure in the *L. monocytogenes* cultures.

Figure 1 shows analysis of *hly* and *clp*C expression under oxidative stress conditions: *hly* expression was upregulated by oxidative stress (in both CHP and H_2_O_2_), and *clp*C was downregulated. ST9 isolated from meat and ST321 only expressed *hly* and *clp*C for the first hour in CHP at 37 °C, and there was a tendency for *hly* downregulation (Figure 1A). 

Strains incubated in CHP showed higher *hly* expression at 10 °C than at 37 °C (Figure 1B). By contrast, *clp*C was downregulated in all strains tested during exposure to CHP regardless of the temperature (37 °C or 10 °C) (Figure 1A,B), with the exception of ST87 that showed *clp*C overexpression after CHP incubation for 3 hours at 10 °C (Figure 1B). We have also studied the relation between *hly* and *clp*C expression during CHP exposure at 10 °C and 37 °C, and it can be observed that *hly* expression was significantly higher when exposed to CHP at 10 °C the 37 °C, whereas *clp*C expression was lower under oxidative conditions regardless of the temperature (Figure 1C).

It was not possible to analyse the gene expression of *hly* and *clp*C in *L. monocytogenes* strains treated with H_2_O_2_ at 37 °C, because *L. monocytogenes* counts were below the detection limit in less than one hour. Only ST87 and ST321 survived exposure to H_2_O_2_ at 10 °C: *hly* was upregulated and *clp*C was downregulated after 3 h (Figure 1D). The relation between *hly* and *clp*C expression during H_2_O_2_ exposure at 10 °C and 37 °C was also studied (Figure 1E): only ST87 and ST321 were able to survive these oxidative conditions, but strains treated with H_2_O_2_ showed *hly* upregulation, while *clp*C was downregulated although its expression was slightly higher at 10 °C.

## 4. Discussion

*L. monocytogenes* is a foodborne bacterium commonly found in food processing plants and is able to withstand adverse conditions [1,11]. In food processing environments (FPE), various stressful conditions can influence *L. monocytogenes* growth and survival, especially refrigeration temperatures, osmotic, acid and oxidative stresses [24,25]. *L. monocytogenes* is also exposed to stressful conditions in hosts and some are common to FPE stresses: gastric acids provide acid and both invasion of phagolysosomes or macrophages and antibiotic treatments can cause oxidative stress [26,27]. *L. monocytogenes* is able to increase its tolerance to stressful conditions over time following repeated exposure to sub-lethal doses. 

We report here that oxidative stress is temperature and oxidizing compound dependent: the effect was significantly lower at lower temperatures (10 °C vs 37 °C), and for CHP than H_2_O_2_. The role of temperature in oxidative stress has also been studied by other authors who reported that lower temperature increased the response to oxidative stress and that there was similar damage to nucleic acids and cell membranes in both stressful conditions [28,29]. In general, detergents and disinfectants (H_2_O_2_, paracetic acid and QAC compounds such as NaOCl, NH_4_OH_4_) used in food industries are applied at refrigeration temperature; this may favour the development in *L. monocytogenes* of resistance to these sanitizer agents over long period time [24]. 

Our comparison of H_2_O_2_ and CHP confirms previous reports showing that H_2_O_2_ is a more effective listericidal agent [27,30]. The presence of molecular oxygen, growth phase and serovar may all affect the response to oxidative conditions [26,31]. From the point of phenotyping strains results, we found that genotypes belonging to STs of Lineage I (ST5, ST6, ST87, ST1) were more resistant to oxidative stress than those of Lineage II (ST7, ST9, ST199 and ST321), with the exception of ST7 that showed tolerance to H_2_O_2_ at 10 °C, however the differences between strains were not statistically significant. This pattern has been observed previously: *L. monocytogenes* serovar 1/2a (Lineage II) is more sensitive than 4b strains (Lineage I) to 0.6 % H_2_O_2_ [24,32].

The differences between lineages may be due to difference in transcription of genes regulating and encoding oxidative responses. *L. monocytogenes* expresses molecular mechanisms based on stress regulator genes (*sig*B, *cts*R, *hrc*A, *lex*A or *rec*A) and response genes (*fri*, *kat*, *per*R or *sod*) against oxidative stress [28,32]. Most of the genes involved in stress responses in *L. monocytogenes* are regulated by *sig*B factor and they include *cts*R, that is the *clp* operon repressor during optimal conditions. Clp family proteins (chaperones and proteases) are generally influenced by temperature [33] and stressful conditions. Likewise, *clp*C is also implicated in the responses to oxidative or high osmolality stresses and iron starvation [13,34]. However, *kat* is frequently considered the most relevant gene oxidative stress response together with *sod*, even at low temperature as Azizoglu & Kathariou (2010) [35] described how *kat* mutant strains showed smaller colonies size and less tolerance to refrigeration or freeze temperatures. In addition, the response to H_2_O_2_ by *kat* could be interfered by the enzymatic reaction from food products [36]. Wherefore, the present study was focused on the expression of *clp*C as representative gene in response to oxidative stress combined with different temperature incubation. It is well known that *L. monocytogenes* virulence is found in island LIPI-1, regulated by *prf*A [37]. Listeriolyin O is encoded by *hly* and its expression could be modified during exposure to range of temperature, osmotic and oxidative environmental conditions [38,39]. However, some studies supported the connection between stress conditions with virulence due to the relation among *sig*B and *prf*A, as *prf*A has three significant promotors dependent of sigA and sigB [14]. This inter-genetic relation could explain why stressed *L. monocytogenes* strains could increase their virulence, although the reaction against the stress could be different depending on possible *prf*A promotor sequence [40].

This study reported that *clp*C was overexpressed in some *L. monocytogenes* strains at 37 °C in the presence of both of oxidizing agents and its expression was downregulated in H_2_O_2_ at 10 °C; these findings implicate *clp*C in the responses to oxidative and heat stresses. Similar results were described by Ochiai et al. [28]. The relationship between stress exposure and virulence in *L. monocytogenes* has been studied previously. Van der Veen and Abee. [13] reported that *clp*C mutant strains (∆*clp*C) can survive inside of macrophages and other host cells; Chastanet et al. [33] found that *clp*P mutants were unable to grow intracellularly; and the promotors of *prf*A (p*Prf*A_1_ and p*Prf*A_2_) and *sig*B (*sig*A and *sig*B) are intrinsically regulated [34]. 

In conclusion, this study describes for the first time the effect of two different oxidizing agents at two temperatures (optimal growth temperature and the refrigeration temperature in food industries) at the same time on different genotypes of *L. monocytogenes.* The oxidative effect is temperature dependent, being lower at 10 °C than 37 °C. The virulence LIPI-1 genes were more strongly expressed when oxidative agents were applied at refrigeration temperatures.

## Figures and Tables

**Table 1:** *L. monocytogenes* log count reduction after exposure to oxidizing agents (CHP and H_2_O_2_) at 37 °C and 10 °C.

				CHP at 37 °C ^a^				CHP at 10 °C				H_2_O_2_ at 10 °C ^b^			
Food Industry	Sample Type	Lineage	Strains	T1	T2	T3	Stn.Error ^c^	T1	T2	T3	Stn.Error	T1	T2	T3	Stn.Error
				Mean	Mean	Mean		Mean	Mean	Mean		Mean	Mean	Mean	
Cheese making factory	Cheese crumbs	I ^d^	ST5 ^f^	5.70	8.35	9.13 *	0.430	0.93	1.43	1.93	0.256	1.15	1.98	1.64	0.212
	Floor	II ^e^	ST7	5.61	7.49	9.05 *	0.611	1.46	2.01	2.36	0.592	0.85	1.54	2.53	0.752
	Cheese crumbs	I	ST6	4.47	7.77	8.95 *	0.148	1.95	1.60	1.96	0.597	1.59	3.47	4.43	0.691
	Cheese crumbs	II	ST9 ^#^	6.75	8.99	9.14 *	0.106	0.75	1.64	2.24	0.237	1.39	3.64	9.15 *	0.184
Meat processing plant	Drain	I	ST87 ^#^	5.02	7.45	8.20	0.831	1.37	2.12	2.77	0.130	0.11	0.68	1.20	0.150
	Boxes	I	ST5 ^#^	5.17	7.57	9.00	0.251	1.19	1.67	2.35	0.098	−0.04	1.85	2.92	0.627
	Floor	II	ST9 ^#^	6.07	8.95	9.25 *	0.171	1.50	1.96	2.60	1.150	3.50	7.15	9.30 *	1.312
	Wall	II	ST9	7.23	9.23 *	9.23 *	0.301	2.09	2.46	3.18	1.061	3.79	9.12 *	9.12 *	0.668
	Floor	I	ST1	4.19	6.08	7.88	0.746	1.88	1.72	1.59	1.037	0.94	2.41	4.74	0.106
	Drain	II	ST199	6.42	7.97	8.71 *	0.621	2.07	2.68	4.07	0.495	3.88	8.62 *	8.62 *	0.114
Seafood company	Water machine	II	ST321 ^#^	7.57	8.92 *	8.92 *	0.679	1.57	3.48	4.43	0.549	0.97	3.71	7.97	0.600

(*) Maximum count reduction; ^a^ Cumene hydroperoxide (CHP); ^b^ Hydrogen peroxide (H_2_O_2_); ^c^ Standard error (SE); ^d^ Lineage I; ^e^ Lineage II; ^f^ Sequence type (ST). ^#^ Strains chosen to gene expression analyses by RT-PCR.
